# One-Year Review of Cardiac Catheterization Procedures at a New Private Cardiac Facility in Nigeria: Indications, Techniques, and Outcomes

**DOI:** 10.7759/cureus.86719

**Published:** 2025-06-25

**Authors:** Olurotimi J Badero, Bamikole Osibowale, Ayobami O Kuyoro, Adedeji Adebayo, Francis C Asogwa, Oyinkansola T Agaja, Loveth C Okonkwo, Olusegun D Alaga, Olutomiwa Omokore

**Affiliations:** 1 Interventional Cardiology, Iwosan Lagoon Hospitals, Victoria Island, Lagos, NGA; 2 Interventional Cardiology, Division of Cardio-Nephrology, Cardiac Renal and Vascular Associates, Jackson, USA; 3 Cardiology, Caribbean Tristate Heart Institute, Trinidad, TTO; 4 Cardiovascular Disease, Iwosan Lagoon Hospitals, Victoria Island, Lagos, NGA; 5 Cardiology, Iwosan Lagoon Hospitals, Ikeja, Lagos, NGA; 6 Internal Medicine, Iwosan Lagoon Hospitals, Ikoyi, Lagos, NGA; 7 Paediatric Cardiology, Iwosan Lagoon Hospitals, Victoria Island, Lagos, NGA; 8 General Practice, Iwosan Lagoon Hospitals, Victoria Island, Lagos, NGA; 9 Cardiology, Iwosan Lagoon Hospitals, Victoria Island, Lagos, NGA; 10 Internal Medicine, Babcock University Teaching Hospital, Ilishan-Remo, NGA; 11 Internal Medicine, Benjamin S. Carson College of Health and Medical Sciences, Ilishan-Remo, NGA

**Keywords:** cardiac catheterization, clinical indications, clinical outcomes, nigeria, private hospital, procedural techniques

## Abstract

Background: Cardiac catheterization is a cornerstone procedure for diagnosing and treating a wide spectrum of cardiovascular diseases globally. This study reports on the establishment and initial outcomes of cardiac and peripheral catheterization services at a newly opened private hospital in Nigeria over a one-year period. The aim is to describe the indications for these procedures, the techniques employed, and the resulting outcomes in this setting.

Methods: A retrospective analysis was conducted on all cardiac and peripheral catheterization procedures performed between the second quarter of 2023 and the second quarter of 2024 at Iwosan Lagoon Hospital. Data regarding patient demographics, the clinical reasons for the procedures, the methods used, and the clinical outcomes were extracted from hospital records and subsequently analyzed and presented as simple frequency tables.

Results: During the study period, a total of 53 procedures were performed. The most frequent reasons for referral were recurrent chest pain and abnormal findings on exercise stress testing. Other indications included cardiomyopathy and claudication. The majority of procedures utilized the femoral artery for vascular access. The overall procedural success rate was 100%, with no major complications reported. A minor complication rate of approximately 3.85% (two complications) was observed.

Conclusion: This initial experience highlights the successful implementation of cardiac catheterization services at a new private facility in Nigeria. The high success rate and low complication rate observed are comparable to international benchmarks. This study contributes valuable data to the growing understanding of cardiac catheterization in the Nigerian context and underscores the importance of ongoing efforts to enhance the quality and accessibility of specialized cardiovascular care in the region.

## Introduction

Cardiac catheterization is a minimally invasive medical procedure essential for the diagnosis and management of various cardiovascular conditions. Since the pioneering self-catheterization by Werner Forssmann in 1929 [1,2], the procedure has become a routine practice worldwide, with over a million performed annually in the United States alone [3].

The establishment of a new cardiac catheterization laboratory at Iwosan Lagoon Hospital in Nigeria represents a significant advancement in the nation's healthcare infrastructure. This private facility was founded with the goal of providing high-quality and more readily accessible cardiac care to the Nigerian population, thereby potentially reducing the need for patients to seek medical treatment abroad.

While diagnostic catheterizations are commonly performed by trained specialists, all such procedures, whether for diagnosis or intervention, carry inherent risks. These risks range from minor issues such as temporary discomfort, nausea, vomiting, bleeding, hematoma, and contrast agent allergies, to more severe complications including myocardial infarction, major embolic events, and even death [4]. Notably, complication rates have significantly decreased in recent years due to advancements in catheter technology and improved operator expertise [5].

This study aims to evaluate the spectrum of clinical indications, the procedural techniques employed, and the clinical outcomes observed during the first year of operation of the cardiac catheterization laboratory at Iwosan Lagoon Hospital in Nigeria.

## Materials and methods

Study design and population

This retrospective study examined all consecutive cardiac and peripheral catheterization procedures performed at Iwosan Lagoon Hospital between June 2023 and July 2024. The analysis included 53 adult patients (age range: 37-79 years) who underwent various diagnostic and interventional procedures, selected through convenience sampling. Procedures comprised left and right heart catheterizations, aortograms, abdomino-femoral run-off peripheral angiographies, percutaneous coronary interventions (PCIs), and peripheral interventions.

Procedural characteristics

The femoral artery served as the primary vascular access site (both right and left sides), with only one case utilizing radial artery access. All procedures adhered to contemporary clinical practice guidelines established by the American College of Cardiology (ACC) and the Society for Cardiovascular Angiography and Interventions (SCAI) [6].

Data collection

We systematically documented all procedure-related complications occurring during hospitalization. Data were extracted from electronic medical records, procedural reports, and nursing documentation. For statistical analysis, we represented data as simple frequencies for both procedures performed and complications observed. This approach provided clear quantification of procedure types and their associated complication rates. The study was approved by the Institutional Review Board at Iwosan Lagoon Hospital.

Statistical analysis

Descriptive analysis was used for continuous variables, and frequency analysis, for categorical variables. Results were expressed as frequencies and percentages. Ages and body mass index (BMI) were presented as means and standard deviations. Data was analyzed using SPSS, version 21.0 (SPSS Inc., Armonk, NY, USA).

Ethical considerations

The study received approval from the hospital's Institutional Review Board, which waived the requirement for informed consent due to the retrospective design. All patient data were anonymized prior to analysis to ensure confidentiality and comply with institutional privacy policies.

## Results

All 53 procedures performed within the defined study period were included in the analysis. The majority were males (39 patients) representing 73.6% of the population and the age range of the population was between 37 and 79 years with a mean age of 60.8 60.8years (Table 1). The distribution of the different types of procedures was as follows: 27 (50.94%) coronary angiographies with ventriculograms, 10 (18.87%) right heart catheterizations, five (9.43%) percutaneous coronary interventions, one (1.89%) aortogram, seven (13.21%) abdomino-femoral run-off angiographies, and three (5.66%) peripheral interventions, including cases of chronic total occlusions (Table 1). The majority of procedures, 50 (94.34%), were performed via the right femoral artery, while two (3.77%) procedures used the left femoral artery, and one (1.89%) utilized the radial artery. Post-procedure haemostasis at the access sites was achieved using various methods in accordance with ACC guidelines, including manual compression, the Mynx femoral closure device, and the radial TR band. The complications associated with all procedures were also documented (Table 1).

**Table 1:** Baseline Characteristics, Procedures and Complications, N (%), * mean and standard deviation

Characteristic	N (%)
Total Number of Patients	53 (100)
Male	39 (73.6)
Mean Age (years)	60.8 (±10.4)*
Body Mass Index (Kg/m2)	27.2 (±3.8)*
Risk Factors	
Dyslipidemia	43 (81.1)
Hypertension	49 (92.5)
Type 2 Diabetes Mellitus	22 (41.5)
Medications	
Angiotensin Converting Enzyme-Inhibitors/Angiotensin Receptor Blockers	51 (96.2)
Calcium Channel Blockers	40 (75.5)
Beta-Blockers	35 (66.0)
Diuretics	15 (28.3)
Antiplatelets	44 (83.0)
Procedures	
Coronary angiogram	27 (50.9)
Right heart catheterization	10 (18.9)
Aortogram	1 (1.9)
Abdomino-femoral run off angiography	7 (13.2)
Percutaneous coronary intervention	5 (9.4)
Percutaneous peripheral intervention	3 (5.7)
Procedural Complications	
Hematoma + hydrocele	1 (1.9)
Vaso-vagal response	1 (1.9)

## Discussion

Since its inception, cardiac catheterization has evolved significantly from a primarily diagnostic tool to encompass a wide range of endovascular interventions for coronary, cerebral, peripheral, and structural heart diseases [7]. The procedures performed at our newly established catheterization laboratory reflect this broad scope.

Diagnostic catheterization

Over the one-year review period, a total of 37 (69.8%) diagnostic catheterization procedures were performed, comprising 27 (50.9%) left heart catheterizations (LHC) and 10 (18.9%) right heart catheterizations (RHC). The most common indications for LHC were unstable angina, while heart failure was the primary indication for RHC. Standard Judkins catheters were typically used for LHC; however, in two cases (3.8%) with challenging coronary anatomy, specialized catheters (Amplatz Left [AL] and Progressive Right [PR]) were required [8]. All diagnostic catheterization procedures in this series were successfully completed without complications. LHC serves both diagnostic and therapeutic purposes, with selective coronary angiography being the gold standard for diagnosing coronary artery disease. Additionally, it is utilized for hemodynamic measurements, assessment of valvular heart disease, percutaneous coronary intervention, closure of congenital cardiac defects, radiofrequency ablation of arrhythmias, and valve replacement [9]. RHC is a crucial diagnostic modality for cardiopulmonary diseases, providing essential data on hemodynamics, cardiac output, and cardiopulmonary pressures that guide clinical decision-making [10]. Definitive diagnoses were established in patients undergoing LHC, while RHC was primarily used to exclude pulmonary hypertension in patients presenting with heart failure.

Percutaneous coronary intervention (PCI)

Percutaneous coronary intervention is a minimally invasive, non-surgical procedure to treat narrowing of the coronary arteries and alleviate cardiac ischemia [9]. Clinical indications for PCI include acute coronary syndromes and angina [11]. The availability of PCI services remains limited in Nigeria, with approximately 12 centers serving a population exceeding 200 million [12]. During the review period, five patients underwent PCI, each requiring the implantation of at least two stents. This represents 19% of the diagnostic LHC procedures performed. All PCI procedures were technically successful and well-tolerated. One patient experienced vasovagal hypotension and bilateral hydrocele secondary to inguinal hematoma as complications following the procedure. Vasovagal syncope in cardiac catheterization is caused by pain and anxiety; it is potentially sinister, especially in patients with pre-existing severe coronary artery disease or valvular stenosis. Consequently, some cardiac catheterization laboratories premedicate with atropine. Also, the tachycardia from atropine may induce angina in patients with severe coronary artery disease [13]. 

Peripheral angiography and peripheral interventions

Peripheral arterial disease (PAD) is a major cause of non-traumatic lower limb amputation [10]. Patients suspected of having PAD were initially evaluated using non-invasive methods such as arterial Doppler ultrasonography [14] and ankle-brachial index [15], followed by peripheral angiography when indicated. Peripheral endovascular interventions aim to restore blood flow in narrowed or blocked peripheral arteries [16]. To date, seven peripheral angiographies have been performed, including one case involving a chronic total occlusion of the superficial femoral artery. All angiographic procedures were successful without complications.

As highlighted above, untreated PAD is a recognized risk factor for limb loss. During the study period, three peripheral interventions were performed, including the revascularization of two superficial femoral arteries with stenting.

Access closure

While manual compression has been the traditional method for achieving hemostasis at vascular access sites, specialized closure devices are now available that facilitate earlier patient ambulation and improve outcomes [17]. At our facility, we have utilized the Mynx closure device [18] in selected patients with favorable results.

Outcomes and complications

All 53 procedures performed during the study period were technically successful. Importantly, there were no major or fatal complications observed.

Challenges

The establishment and operation of a new cardiac catheterization laboratory in our setting present several challenges. Firstly, there is a scarcity of adequately trained interventional cardiologists and allied healthcare professionals, including nurses and technicians with expertise in cardiac catheterization procedures [19]. Ensuring adequate training and retention of skilled personnel remains a significant hurdle. Secondly, financial constraints pose a major limitation. Medical insurance coverage in Nigeria often does not include cardiac catheterization procedures, making the cost prohibitive for many patients [20]. Thirdly, maintaining a consistent supply of essential consumables, such as catheters, stents, and other specialized medical devices, can be challenging. The importation of these items can be expensive and subject to logistical delays. Lastly, there is a need to increase public awareness regarding the availability of cardiac catheterization services and the importance of early diagnosis and treatment of cardiovascular conditions that can be effectively managed with minimally invasive procedures.

Future plans and development

The organization plans to commence acute cases management, expand the range of diagnostic and interventional services offered. Additionally, there is a plan to upgrade existing equipment and facilities. Furthermore, there is a plan to implement comprehensive staff training and professional development programs.

## Conclusions

This initial one-year review of cardiac and peripheral catheterization procedures performed at a new private facility in Nigeria demonstrates the successful establishment of these crucial services. The key findings highlight a high procedural success rate and a low incidence of complications, which are encouraging and compare favorably with international standards. This study provides valuable insights into the indications, techniques, and outcomes of cardiac catheterization in this developing healthcare environment. Moving forward, continued efforts focused on quality improvement, staff training, and addressing the existing challenges will be essential to further enhance the provision of advanced cardiovascular care in Nigeria.
